# Editorial: Pathway to recovery: understanding the plastic changes in neural circuits leading to recovery

**DOI:** 10.3389/fnins.2024.1411938

**Published:** 2024-04-24

**Authors:** Mesut Sahin, Sean K. Meehan, George C. McConnell

**Affiliations:** ^1^Department of Biomedical Engineering, New Jersey Institute of Technology, Newark, NJ, United States; ^2^Department of Kinesiology and Health Sciences, University of Waterloo, Waterloo, ON, Canada; ^3^Department of Biomedical Engineering, Stevens Institute of Technology, Hoboken, NJ, United States

**Keywords:** neural plasticity, spinal cord injury, neural regeneration, neuromodulation, cerebellum

The nervous system has the ability to reorganize and adapt to challenges following injury. However, harnessing this remarkable potential for plasticity to facilitate functional recovery is a challenge. Immediately after injury, acute stressors promote structural and functional reorganization that may interact with subsequent experience-dependent processes to shape plastic potential. The articles assembled under this Research Topic represent a multidisciplinary approach to understanding how the nervous system responds to injury and how we can maximize adjunctive therapies and assistive technologies to promote sensorimotor recovery and maximize quality of life. These articles generally fall into three clusters. One cluster seeks to quantify changes in sensorimotor control following traumatic peripheral or spinal injury. A second cluster investigates the benefits of neural modulation targeting the spinal cord and cortex. A final cluster focuses on the feasibility of emerging methods to quantify sensorimotor plasticity.

Several papers in this Research Topic highlight the complexity of the central nervous system's response to traumatic injury. For example, the papers by Zheng et al. and Cetinkaya et al. draw our attention to the importance of cerebello-cerebral connections following spinal cord injury (SCI). The cerebellum is a critical integrative center. For instance, the paper by Cetinkaya et al. presents evidence that the cerebellum makes its contribution, particularly at the initiation and termination phases of the forelimb-reaching behavior in the rat model. Interestingly, the high-frequency components in the multi-unit activity band, rather than the local field potentials, of the cerebellar cortical activity had higher correlations with the forelimb muscle activity in these phases. The cerebellum's reciprocal loops with the spinal cord, sensory, motor and cognitive cortices play key roles in coordinating skilled actions from reaching to balance. Therefore, it should not be surprising that Zheng et al. demonstrate reduced cerebello-cerebral functional connectivity that spans sensorimotor, auditory, visual and cognitive substrates following complete thoracolumbar injury. Interestingly, Zheng et al. identify projections between lobule 10 of the cerebellar vermis and the fusiform gyrus, a higher-order visual area, as a potential target for neuromodulation to enhance sensorimotor ability post-injury. In addition to changes in cerebello-cerebral connectivity, Torres et al. demonstrate that sensorimotor plastic change following traumatic brachial plexus injury (TBPI) is not restricted to the cortical representations of the injured limb. Using afferent inhibition, a measure of sensorimotor integration, Torres et al. observed typical sensorimotor integration in the first dorsal interosseous sensorimotor motor cortex. However, heterotopic cutaneous stimulation of the lip was atypical following TBPI. Post-injury adaptations are also observed in the oscillatory properties of sensorimotor neurons. For example, Shan et al. report significantly greater oscillatory activity in central sensorimotor areas coupled with decreases in oscillatory activity in frontal, precentral and postcentral brain regions during lower limb motor imagery following left lower limb amputation. Correlations between sensorimotor beta power during motor imagery and resting state functional connectivity led Shan et al. to hypothesize that increased contralateral beta power during motor imagery may compensate for remodeled connectivity in sensorimotor networks responsible for amputated limb control.

A couple of papers in this Research Topic focus on the potential for non-invasive neuromodulation at different levels of the nervous system as an adjunctive therapy to enhance function following injury. Parhizi et al. investigated the potential of transcutaneous spinal cord stimulation (tSCS) to improve upper and lower limb coordination during locomotion in healthy participants. Although tSCS effects on these intraspinal connections remain to be seen in SCI patients, this study points out the importance of multi-point stimulation for inducing neuroplastic effects in the spinal cord. The paper by Katagiri et al. highlights the challenge of using cortical non-invasive brain stimulation to probe plasticity mechanisms and its potential as an adjunctive treatment. Group-level after-effects in the tibialis anterior following facilitatory or inhibitory theta burst stimulation were highly variable across participants. Heterogeneity in the induced neuroplastic after-effect highly depended on the individual's baseline cortical excitability and intracortical network state. The work by Katagiri et al. extends similar observations in the upper limb and illustrates the need for an enhanced understanding of individual predictors of patterned, repetitive stimulation responses.

This Research Topic's final cluster of papers focuses on the feasibility of methodologies used to evaluate sensorimotor function and quantify plastic changes in the nervous system. At the functional level, Heinzel et al. aimed to determine the extent to which computerized gait analysis is a valid method to evaluate functional recovery following autograft repair of the rat median nerve. Correlation analysis between well-established measures of motor and sensory recovery gait parameters identified parameters such as Print Area, Duty Cycle and Stand Index that could be used to assess nerve regeneration. Although functional assessments can provide valuable markers of recovery, such measures likely represent many different mechanistic influences. As papers in this Research Topic establish, there can be changes in function driven by plasticity in cortical, subcortical, or spinal systems and their interactions. Access to ascending and descending signals at various levels of the nervous system can provide mechanistic insights that functional biomarkers cannot. Neural recordings in the spinal cord are challenging because of the neural trauma induced by the electrodes in a moving spinal cord. However, Fathi and Erfanian. demonstrate the feasibility of using local field potentials recorded directly from the dorsal and lateral columns of the spinal cord to decode hindlimb kinematics during locomotion in a cat model. The onset and offset of hindlimb movement were clearly decoded by spinal event-related synchronizations and desynchronizations across frequency bands, while spinal theta power was correlated with kinematics such as locomotory speed.

It is our expectation that the fundamental findings across this diverse assemblage of papers will prompt cross disciplinary collaborations. We hope that new perspectives driven by collaboration will dismantle barriers to progress and accelerate the development of new approaches and technologies for those whose quality of life is impacted by diseases or injuries that impact nervous system function.

## Author contributions

MS: Writing – original draft, Writing – review & editing. SM: Writing – original draft, Writing – review & editing. GM: Writing – original draft, Writing – review & editing.

